# Supplementary figures and images for: Optimised genome editing for precise DNA insertion and substitution using prime editors in zebrafish
Source: eLife. 2026 Jul 7;14:RP107475. doi: 10.7554/eLife.107475 (PMC13341114; doi:10.7554/eLife.107475)

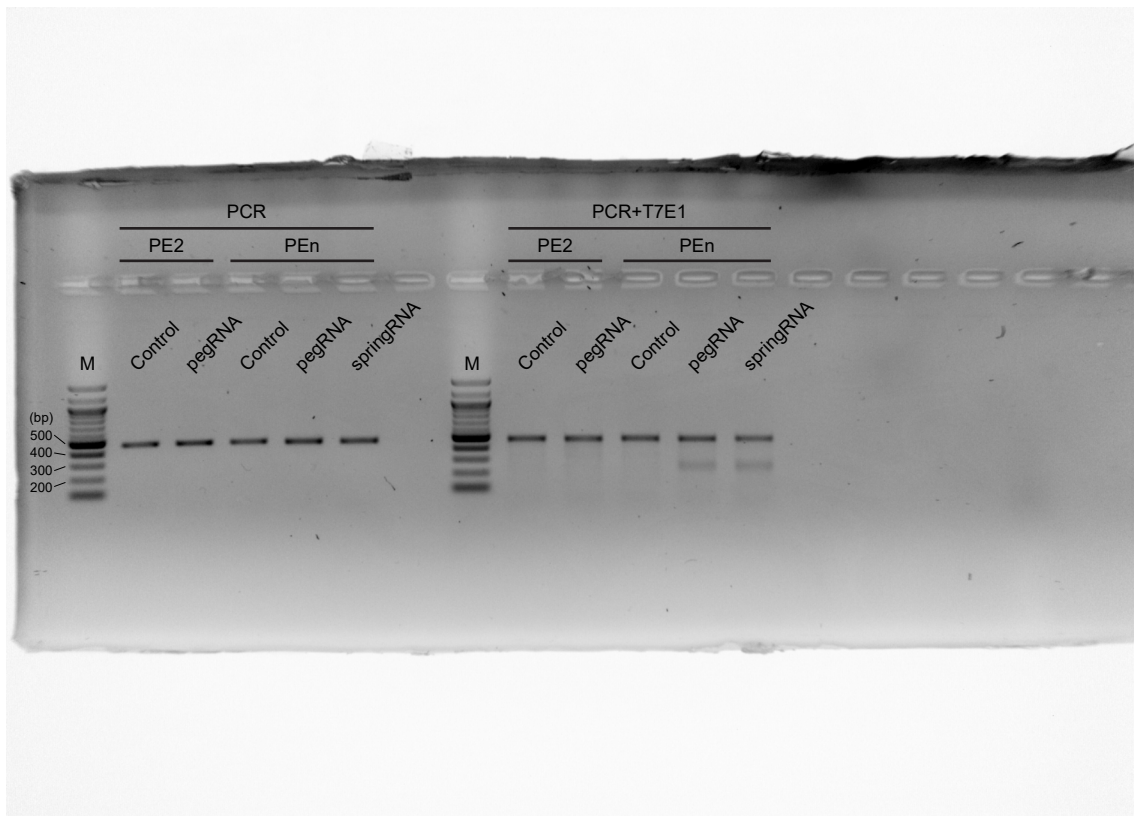

Figure 2, Source data 1. Original agarose gel image corresponding to Figure 2, panel D. M, DNA marker.

Supplement: Figure 2—source data 1. [file elife-107475-fig2-data1.zip › Figure 2_Source data 1.pdf]

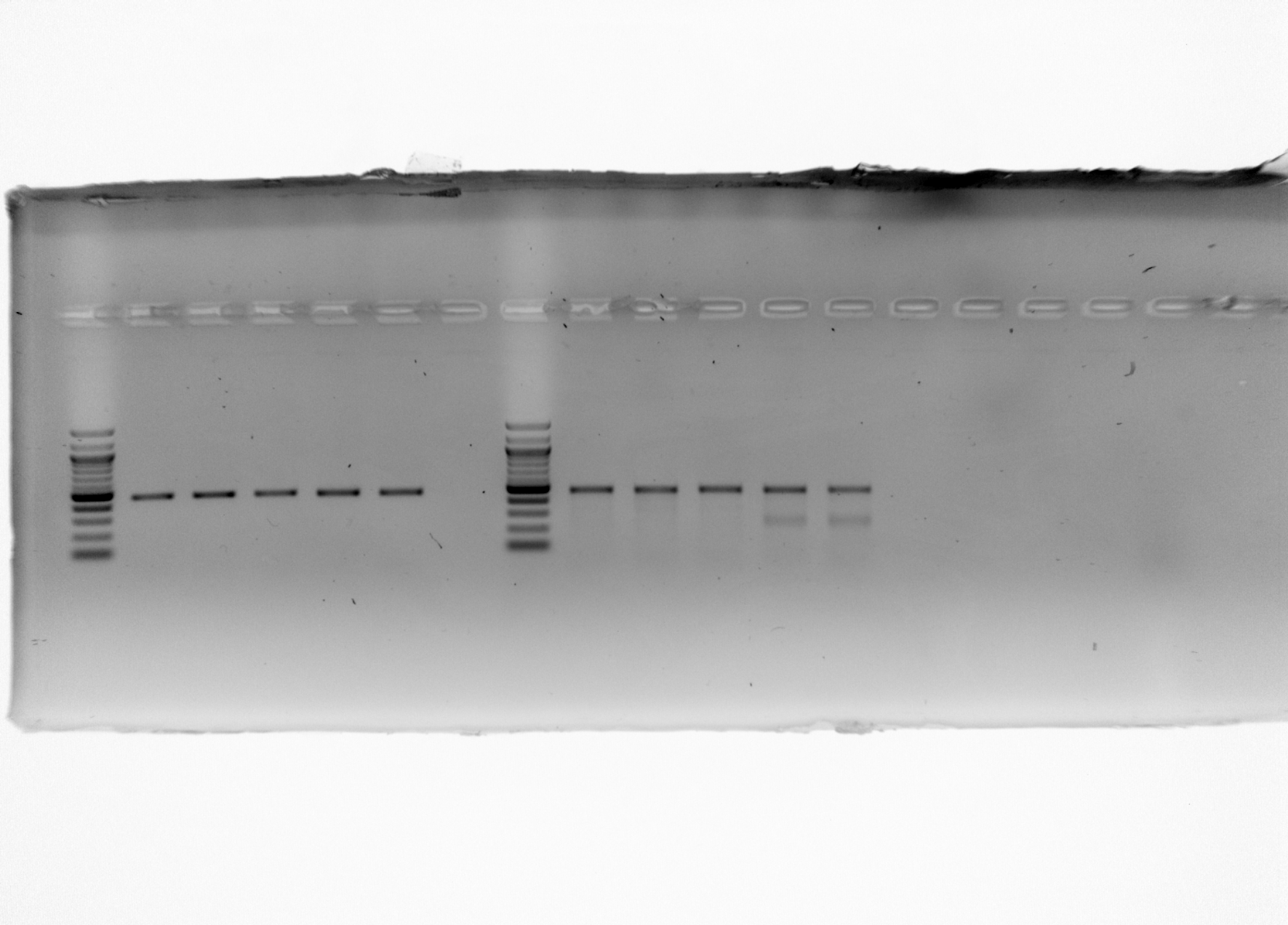

Supplement: Figure 2—source data 2. [file elife-107475-fig2-data2.zip › ror2 exon9 PCR&T7E1.tif]
