# Supplementary material for: Optimised genome editing for precise DNA insertion and substitution using prime editors in zebrafish
Source: eLife. 2026 Jul 7;14:RP107475. doi: 10.7554/eLife.107475 (PMC13341114; doi:10.7554/eLife.107475)
Supplement: MDAR checklist [file elife-107475-mdarchecklist1.docx]

**Materials Design Analysis Reporting (MDAR)**

**Checklist for Authors**

The [MDAR framework](https://osf.io/xfpn4/) establishes a minimum set of requirements in transparent reporting mainly applicable to studies in the life sciences.

*eLife* asks authors to **provide detailed information within their article** to facilitate the interpretation and replication of their work. Authors can also upload supporting materials to comply with relevant reporting guidelines for health-related research (see [EQUATOR Network](http://www.equator-network.org/%20)), life science research (see the [BioSharing Information Resource](http://biosharing.org/)), or animal research (see the [ARRIVE Guidelines](http://www.plosbiology.org/article/info:doi/10.1371/journal.pbio.1000412) and the [STRANGE Framework](https://doi.org/10.1038/d41586-020-01751-5); for details, see *eLife*’s [Journal Policies](https://reviewer.elifesciences.org/author-guide/journal-policies)). Where applicable, authors should refer to any relevant reporting standards materials in this form.

For all that apply, please note **where in the article** the information is provided. Please note that we also collect information about data availability and ethics in the submission form.

**Materials:**

| **Newly created materials** | **Indicate where provided: section/figure legend** | **N/A** |
| --- | --- | --- |
| The manuscript includes a dedicated "materials availability statement" providing transparent disclosure about availability of newly created materials including details on how materials can be accessed and describing any restrictions on access. | The information on genetically modified zebrafish created in the study will be indexed in Zebrafish Information Network community website (https://zfin.org/) after the publication of the article. |  |
|  |  |  |
| **Antibodies** | **Indicate where provided: section/figure legend** | **N/A** |
| For commercial reagents, provide supplier name, catalogue number and [RRID](https://scicrunch.org/resources), if available. |  | N/A |
|  |  |  |
| **DNA and RNA sequences** | **Indicate where provided: section/figure legend** | **N/A** |
| Short novel DNA or RNA including primers, probes: Sequences should be included or deposited in a public repository. | Sequences of primers, guide RNAs, and donor DNAs are listed in Materials and Methods section and Supplementary Tables. |  |
|  |  |  |
| **Cell materials** | **Indicate where provided: section/figure legend** | **N/A** |
| Cell lines: Provide species information, strain. Provide accession number in repository OR supplier name, catalog number, clone number, OR RRID. |  | N/A |
| Primary cultures: Provide species, strain, sex of origin, genetic modification status. |  | N/A |
|  |  |  |
| **Experimental animals** | **Indicate where provided: section/figure legend** | **N/A** |
| Laboratory animals or Model organisms: Provide species, strain, sex, age, genetic modification status. Provide accession number in repository OR supplier name, catalog number, clone number, OR RRID. | Zebrafish wild-type strain and transgenic allele information are stated with reference in Materials and Methods section. |  |
| Animal observed in or captured from the field: Provide species, sex, and age where possible. |  | N/A |
|  |  |  |
| **Plants and microbes** | **Indicate where provided: section/figure legend** | **N/A** |
| Plants: provide species and strain, ecotype and cultivar where relevant, unique accession number if available, and source (including location for collected wild specimens). |  | N/A |
| Microbes: provide species and strain, unique accession number if available, and source. | Product name and company information of commercial competent cells are stated in Materials and Methods section. |  |
|  |  |  |
| **Human research participants** | **Indicate where provided: section/figure legend) or state if these demographics were not collected** | **N/A** |
| If collected and within the bounds of privacy constraints report on age, sex, gender and ethnicity for all study participants. |  | N/A |

**Design:**

| **Study protocol** | **Indicate where provided: section/figure legend** | **N/A** |
| --- | --- | --- |
| If the study protocol has been pre-registered, provide DOI. For clinical trials, provide the trial registration number OR cite DOI. |  | N/A |
|  |  |  |
| **Laboratory protocol** | **Indicate where provided: section/figure legend** | **N/A** |
| Provide DOI OR other citation details if detailed step-by-step protocols are available. | Citation details of the step-by-step protocol for Prime Editor protein expression and purification (Antoniou et al., 2025) are stated in References section. |  |
|  |  |  |
| **Experimental study design (statistics details) *** | | |
| **For in vivo studies: State whether and how the following have been done** | **Indicate where provided: section/figure legend. If it could have been done, but was not, write “not done”** | **N/A** |
| Sample size determination | Sample size was determined using G*Power software with thresholds of p < 0.05 and power of 0.80–0.95, and based on that in previous publications. The sample size (number of animals analysed) in each analysis was stated in Figures and Figure legends. |  |
| Randomisation | In microinjection experiment, zebrafish embryos collected from at least three independent pairs and the samples for analysis were randomly selected from 50-60 injected animals. The details of sampling and the sample number used for each analysis are stated in the main text, Materials and Methods section, and Figure legends. |  |
| Blinding |  | N/A |
| Inclusion/exclusion criteria |  | N/A |
|  |  |  |
| **Sample definition and in-laboratory replication** | **Indicate where provided: section/figure legend** | **N/A** |
| State number of times the experiment was replicated in the laboratory. | In vivo experiments (microinjection) were performed individually on zebrafish embryos collected from at least three independent pairs. The number of animals analysed in each analysis was stated in Figures and Figure legends. |  |
| Define whether data describe technical or biological replicates. | Unit of the study (zebrafish embryo) was defined in the main text and Figure legends. |  |
|  |  |  |
| **Ethics** | **Indicate where provided: section/submission form** | **N/A** |
| Studies involving human participants: State details of authority granting ethics approval (IRB or equivalent committee(s), provide reference number for approval. |  | N/A |
| Studies involving experimental animals: State details of authority granting ethics approval (IRB or equivalent committee(s), provide reference number for approval. | Details of the authorities granting experimental procedure and ethics approval are stated in Materials and Methods section with the UK Home Office license number. |  |
| Studies involving specimen and field samples: State if relevant permits obtained, provide details of authority approving study; if none were required, explain why. |  | N/A |
|  |  |  |
| **Dual Use Research of Concern (DURC)** | **Indicate where provided: section/submission form** | **N/A** |
| If study is subject to dual use research of concern regulations, state the authority granting approval and reference number for the regulatory approval. |  | N/A |

**Analysis:**

| **Attrition** | **Indicate where provided: section/figure legend** | **N/A** |
| --- | --- | --- |
| Describe whether exclusion criteria were pre-established. Report if sample or data points were omitted from analysis. If yes, report if this was due to attrition or intentional exclusion and provide justification. | Where a sample was excluded from analysis, the sample information and the justification are stated in the Figure legends (Figure 3 and 5). |  |
|  |  |  |
| **Statistics** | **Indicate where provided: section/figure legend** | **N/A** |
| Describe statistical tests used and justify choice of tests. | Statistical tests used in the article and the criteria for the choice are stated in Materials and Methods section. |  |
|  |  |  |
| **Data availability** | **Indicate where provided: section/submission form** | **N/A** |
| For newly created and reused datasets, the manuscript includes a data availability statement that provides details for access (or notes restrictions on access). | Data availability section in the manuscript states the details for access to the dataset. Source Data files were provided with the manuscript. |  |
| When newly created datasets are publicly available, provide accession number in repository OR DOI and licensing details where available. |  | N/A |
| If reused data is publicly available provide accession number in repository OR DOI, OR URL, OR citation. |  | N/A |
|  |  |  |
| **Code availability** | **Indicate where provided: section/figure legend** | **N/A** |
| For any computer code/software/mathematical algorithms essential for replicating the main findings of the study, whether newly generated or re-used, the manuscript includes a data availability statement that provides details for access or notes restrictions. | Software used to analyse the data in the article are listed with version information in Materials and Methods section. Parameters of CRISPResso analysis are listed in Supplementary Table 3. |  |
| Where newly generated code is publicly available, provide accession number in repository, OR DOI OR URL and licensing details where available. State any restrictions on code availability or accessibility. |  | N/A |
| If reused code is publicly available provide accession number in repository OR DOI OR URL, OR citation. |  | N/A |

**Reporting:**

The MDAR framework recommends adoption of discipline-specific guidelines, established and endorsed through community initiatives.

| **Adherence to community standards** | **Indicate where provided: section/figure legend** | **N/A** |
| --- | --- | --- |
| State if relevant guidelines (e.g., ICMJE, MIBBI, ARRIVE, STRANGE) have been followed, and whether a checklist (e.g., CONSORT, PRISMA, ARRIVE) is provided with the manuscript. |  | N/A |

* We provide the following guidance regarding transparent reporting and statistics; we also refer authors to [Ten common statistical mistakes to watch out for when writing or reviewing a manuscript](https://doi.org/10.7554/eLife.48175).

**Sample-size estimation**

- You should state whether an appropriate sample size was computed when the study was being designed
- You should state the statistical method of sample size computation and any required assumptions
- If no explicit power analysis was used, you should describe how you decided what sample (replicate) size (number) to use

**Replicates**

- You should report how often each experiment was performed
- You should include a definition of biological versus technical replication
- The data obtained should be provided and sufficient information should be provided to indicate the number of independent biological and/or technical replicates
- If you encountered any outliers, you should describe how these were handled
- Criteria for exclusion/inclusion of data should be clearly stated
- High-throughput sequence data should be uploaded before submission, with a private link for reviewers provided (these are available from both GEO and ArrayExpress)

**Statistical reporting**

- Statistical analysis methods should be described and justified
- Raw data should be presented in figures whenever informative to do so (typically when N per group is less than 10)
- For each experiment, you should identify the statistical tests used, exact values of N, definitions of center, methods of multiple test correction, and dispersion and precision measures (e.g., mean, median, SD, SEM, confidence intervals; and, for the major substantive results, a measure of effect size (e.g., Pearson's r, Cohen's d)
- Report exact p-values wherever possible alongside the summary statistics and 95% confidence intervals. These should be reported for all key questions and not only when the p-value is less than 0.05.

**Group allocation**

- Indicate how samples were allocated into experimental groups (in the case of clinical studies, please specify allocation to treatment method); if randomization was used, please also state if restricted randomization was applied
- Indicate if masking was used during group allocation, data collection and/or data analysis
