# Supplementary material for: Optimised genome editing for precise DNA insertion and substitution using prime editors in zebrafish
Source: eLife. 2026 Jul 7;14:RP107475. doi: 10.7554/eLife.107475 (PMC13341114; doi:10.7554/eLife.107475)
Supplement: Peer review history

# Editor report: eLife Assessment

**DOI:** 10.7554/eLife.107475.3.sa0

**Authors:** Filippo Del Bene

**Affiliations:** https://ror.org/000zhpw23 Institut de la Vision France

**Keywords:** Convincing, Valuable

This study presents a comparison of the efficiency and precision of two prime editing methods to introduce single-nucleotide variants and longer exogenous DNA sequences into the zebrafish genome. **Convincing** data support the conclusion that the PE2 prime editor Nickase is more effective at introducing single-nucleotide variants, while the PEn prime editor nuclease is more effective at integrating sequences from 3 up to 46 base pairs, for both somatic and germline editing. The results will be **valuable** for the zebrafish community, in particular to model human disease variants in this model organism.

# Referee report: Reviewer #1 (Public review)

**DOI:** 10.7554/eLife.107475.3.sa1

Ono et al., compared the activity of prime editor nickase PE2 and primer editor nuclease PEn in introducing SNPs and short exogenous DNA sequences into the zebrafish genome to model human disease variants. They find the nickase PE2 prime editor had a higher rate of precise integration for introducing single nucleotide substitutions, whereas the nuclease PEn prime editor showed improved precision of integration of short DNA sequences. In somatic tissue the percentage of SNP variant precision edits improved when using PE2 RNP injection instead of mRNA injection, but increased precision editing correlated with elevated indel formation. While PEn overall had higher rates of precision edits, the indel rate was also elevated. Similar rates were observed when introducing a 3 bp stop codon into the ror gene using a standard pegRNA with a 13-nucleotide homology arm, or a springRNA driving integration by NHEJ. Inclusion of an abasic sequence in the springRNA prevented imprecise edits caused by scaffold incorporation, but did not improve the overall percentage of precise edits in somatic tissue. Both PE2 and PEn showed higher frequency of 3 bp precision integration, compared to CRISPR HDR mediated knock-in using a single strand donor DNA template with short homology. Recovery of a germline ror-TGA integration allele using PEn with RNP was robust, resulting in 5 out of 10 founders transmitting a precise allele. The authors demonstrate PEn was effective at integration of a 30 bp nuclear localization signal into the 5' end of GFP in an existing muscle-specific reporter line. PEn-mediated integration of long sequences was further demonstrated by integration into the wls gene of a 46bp attP sequence for phiC31 integrase recombination. Additional analyses are needed to determine if the approach can be used to isolate stable germline alleles of variants that are potentially dominant negative or gain of function in nature.

The conclusions of the paper are well supported, demonstrating PE2 increases precision, while PEn increases efficiency, for integrating short DNA sequences. Introducing longer sequences up to 46 bp wit PEn highlights the potential broad utility of this approach for insertion of functional motifs for protein modification and gene expression.

(1) In Figure 3 the data indicates a significant increase in precise edits of the 3 bp TGA using PE2 RNP (11.5%) vs. PE2 mRNA (1.3%). At the adgrf3b locus both PE2 RNP, PE2 mRNA, PEn RNP and PEn mRNA were tested for introducing the 3 bp TGA and a longer 12 bp insertion. PEn RNP showed the highest rate of precision for integration of the longer 12 bp sequence. A comparison of somatic precision editing at additional loci, and analysis of germline transmission rates using PE2 vs. PEn, would support the conclusion that PEn is preferred for precise integration of longer templates, and recovery of germline integration alleles.

(2) Figure 4 shows the results of introducing a TGA stop codon that is predicted to result in nonsense mediated decay. Testing the ability to also isolate different substitution mutations in the germline would be useful information for identifying the most effective approach for generating human disease variant models.

# Referee report: Reviewer #2 (Public review)

**DOI:** 10.7554/eLife.107475.3.sa2

The manuscript by Ono et al compares two prime editing strategies in zebrafish, one based on a nickase and the other on a nuclease, and evaluates their performance for introducing substitutions, short insertions, and transmission to the next generation. The study aims to clarify the relative strengths of these approaches and to extend their use for inserting short DNA sequences in vivo.

The study provides a useful and well-executed comparison of two editing strategies in a vertebrate model. In particular, the finding that the nuclease-based approach shows higher efficiency for short insertions is of practical interest for functional studies. The authors also present convincing evidence supporting their conclusions, including sequencing and phenotypic validation at selected loci. These results support the reliability of the approach in this system.

The overall conceptual advance remains somewhat limited, as the general strategy of delivering prime editing components in zebrafish has been described previously. The present study extends this work by comparing two editing modes and exploring insertion efficiency, which represents a useful but incremental advance.

Regarding the comparison between the two systems, the authors have made efforts to address concerns about generalizability by adding data from additional loci and by refining the scope of their conclusions. These additions strengthen the manuscript. However, the comparison is still based on a relatively small number of loci, and the conclusions may therefore remain somewhat context-dependent.

Overall, the authors largely achieve their stated aims of comparing two editing strategies and demonstrating their applicability in zebrafish. The data generally support the conclusions, particularly within the tested loci. The work provides practical value to the community, especially for researchers seeking efficient strategies for short sequence insertion in this model system, although its broader impact is somewhat limited by its incremental nature.

# Referee report: Reviewer #3 (Public review)

**DOI:** 10.7554/eLife.107475.3.sa3

The manuscript by Ono et al describes application of prime editors to introduce precise genetic changes in the zebrafish model system. Probably the most important observation is that compared to the "standard" PE2, prime editor with full nuclease activity appears to be more efficient at introducing insertions into the genome. Although many laboratories around the world have successfully used oligonucleotide-mediated HDR to insert short exogenous sequences such as epitope tags or loxP sites into the zebrafish genome, the method suffers from high frequency of indels at the edit site. Thus, additional tools are badly needed, making this manuscript very important.

Comments on revised version.

Thank you for thoroughly addressing my minor concerns.

# Author comment: Author response

**DOI:** 10.7554/eLife.107475.3.sa4

**Authors:** Yosuke Ono, Martin Peterka, Michael Love, Amir Khan, Felix Bowers, Ashish Bhandari, Euan Gordon, Jonathan S Ball, Chrissy Hammond, Charles R Tyler, Steve Rees, Mohammad Bohlooly-Y, Marcello Maresca, Steffen Scholpp

**Affiliations:** University of Exeter Exeter United Kingdom; AstraZeneca Gothenburg Sweden; University of Exeter Exeter United Kingdom; School of Physiology, Pharmacology and Neuroscience, University of Bristol Bristol United Kingdom; School of Physiology, Pharmacology and Neuroscience, University of Bristol Bristol United Kingdom; University of Exeter Exeter United Kingdom; AstraZeneca Gothenburg Sweden; University of Exeter Exeter United Kingdom; School of Physiology, Pharmacology and Neuroscience, University of Bristol Bristol United Kingdom; University of Exeter Exeter United Kingdom; https://ror.org/04r9x1a08 AstraZeneca Cambridge United Kingdom; AstraZeneca Gothenburg Sweden; AstraZeneca Gothenburg Sweden; University of Exeter Exeter United Kingdom

The following is the authors’ response to the original reviews.

Thank you very much for handling our revised manuscript and for the careful and constructive comments from the reviewers. We are grateful for the detailed feedback, which has helped us improve both the experimental presentation and the framing of the study. In response to the comments, we have substantially revised the manuscript, updated the figures and supplementary figures, and clarified several points in the text. We have also added new experimental analyses, which were essential to strengthen the manuscript.

We would like to highlight the major changes in the revised version:

Added the late phenotype analysis of the ror2 mutant, including loss of nasal and maxillary barbels and altered adult jaw morphology by microCT, strengthening the disease-model relevance.

Added new data on a further target locus (*wls*) showing 46 bp attP insertion by PEn and comparison with HDR-mediated knock-in at the same site.

Expanded the analysis of insertion performance at *adgrf3b* and clarified comparison with previously reported PE2 data.

Added the analysis of HDR-mediated knock-in and prime editing substitution to generate *ror2* W722X allele.

Added comparative off-target analysis for PE2, PEn and HDR at three predicted off-target sites for the *ror2* target.

Resolved the cloning/NGS inconsistency for *ror2* by increasing clone analysis

We have also moderated several statements in the manuscript, for example, that editing efficiency is locus- and edit-dependent, and that broader comparison of germline transmission efficiencies between prime editing systems will require future work.

A few reviewer suggestions would have required substantial additional experimental work that is technically demanding and beyond the immediate scope of the present methods-focused resubmission, for example, a direct side-by-side germline comparison of PE2 and PEn across several loci, or systematic cost benchmarking against HDR across multiple edit classes. Rather than overstate these points, we have acknowledged these limitations directly in the revised manuscript and narrowed our claims accordingly.

**Public Reviews:**

**Reviewer #1 (Public review):**

From the work presented, it is unclear how prime editing could be used to transiently model human pathogenic variants, given the low frequency of precision edits in somatic tissue, or to isolate stable germline alleles of variants that are potentially dominant negative or gain-of-function in nature. Without a direct comparison with CRISPR/Cas9 nuclease HDR-based methods that use oligonucleotide templates to introduce edits, the advantage of prime editing is unclear. A cost comparison between prime editing and HDR methods would also be of interest, particularly for integration of longer DNA sequences

We thank the reviewer for this important comment. In response, we added a direct comparison between PEn-mediated editing and HDR-mediated knock-in at the *ror2* locus and the *wls* locus using insertion of a 46 bp attP sequence. This new dataset shows that PEn can achieve programmed insertion at a higher efficiency in *ror2* and comparable efficiency in *wls* to HDR at the same target site, thereby providing a more direct benchmark within zebrafish embryos. We also revised the Discussion to better position prime editing as a practical donor DNA-free approach rather than as a universally superior method. We agree that a formal cost comparison would be informative; however, such an analysis would depend strongly on locus, edit size, optimisation burden, and local reagent production pipelines, and we believe this is beyond the scope of the present manuscript. Instead, we now discuss these practical considerations more cautiously in the revised Discussion.

(1) In Figure 3, the data indicate a significant increase in precise edits of the 3 bp TGA using PE2 RNP (11.5%) vs. PE2 mRNA (1.3%). At the adgrf3b locus, only PEn mRNA was tested for introducing the 3 bp and 12 bp insertions. The previous study testing PE2 for 3 and 12 bp insertions was mentioned, but the frequency was not listed, and the study wasn't cited (lines 204 - 207). A comparison of germline transmission rates using PE2 vs. PEn would support the conclusion that PEn allows precise integration of longer templates and recovery of germline integration alleles.

We appreciate this point. We revised the *adgrf3b* section to include the relevant reference and explicitly state the previously reported PE2 frequencies, allowing clearer comparison with our PEn data. We added our own experimental data to compare PE2 and PEn with mRNA or RNP form in *adgrf3b* locus (Figure 3I and J). We also refined the wording of our conclusions so that we do not imply a direct germline comparison between PE2 and PEn where such data are not available. In the revised manuscript, we now state that our germline transmission results apply to PEn-mediated insertions in the loci tested here. A full side-by-side germline comparison between PE2 and PEn across multiple loci would indeed be valuable, but this would require substantial additional animal work and time and is beyond the scope of the present resubmission.

(2) Figure 4 shows the results of introducing a TGA stop codon that is predicted to result in nonsense-mediated decay. Testing the ability to also isolate different substitution mutations in the germline would be useful information for identifying the most effective approach for generating human disease variant models.

We agree that this would be useful. In the present study, we focused experimentally on establishing stable lines for the insertion-based edits, while the substitution experiments were used to compare PE2 and PEn performance in somatic editing at the crbn locus. We also tested the generation of *ror2* W722X allele by prime editing substitution (Figure 3—figure supplement 1). We have therefore revised the manuscript to clarify the scope of the disease-modelling claim and now state more explicitly that our data support the generation of disease-relevant alleles in cases where short, programmed substitutions or insertions are sufficient.

A comparison with the prime editing variant knock-in frequencies reported in the recent publication by Vanhooydonck et al., 2025, Lab Animal should be included in the Discussion.

We have added this study to the revised manuscript and now discuss our findings in relation to the frequencies reported by Vanhooydonck et al. (2025).

**Reviewer #2 (Public review):**

The comparative analysis between PE2 and PEn systems suffers from limited evidentiary support. The comparison relies on single loci for substitutions (crbn) and insertions (ror2), raising concerns about generalizability. Additional validation across multiple loci is necessary to support broad conclusions about PE2/PEn performance

We appreciate this concern. To strengthen the manuscript, we added new experimental data at an additional target locus, *wls*, where we tested insertion of a 46 bp attP sequence and compared PEn with HDR-mediated knock-in. We also included the *adgrf3b* insertion data more prominently. At the same time, we revised the wording throughout the manuscript so that our conclusions are more carefully limited to the loci tested here.

**Reviewer #3 (Public review):**

(1) The logic for introducing two nucleotide changes (at +3 and +10) to change a single amino acid (I378) should be explicitly explained in the main body of the manuscript. It is indeed self-explanatory when looking at Supplementary Figure 1. One way of doing it could be to include Supplementary Figure 1a in Figure 1.

We thank the reviewer for pointing this out. We have now explained this directly in the main text. Specifically, we state that one nucleotide change introduces the desired missense mutation, whereas the second was included to reduce potential pegRNA misfolding caused by complementarity between the spacer and the PBS/RT template region.

(2) It is not clear why a 3-nucleotide insertion was used to generate W722X. The human W720X is a single-nucleotide polymorphism, and it should be possible to make a corresponding zebrafish mutant by introducing two nucleotide changes.…

We agree that this point and have now explained in the main text that the 3 bp stop-codon insertion was chosen as a proof-of-principle strategy for generating a precisely truncated protein through programmed insertion, a type of edit that can be broadly applied to target loci. We also tested the generation of *ror2* W722X allele by prime editing substitution (Figure 3—figure supplement 1). We also clarify that prime editing substitution was tested separately here.

(3) Lines 137-138: T7 Endonuclease assay used in Figure 2d detects all polymorphisms, both precise changes and indels. Thus, if this assay were performed on embryos shown in Figure 1c-d, the overall percentage of modified alleles would be similarly higher for PEn over PE2 (add up precise prime edits and indels). The conclusion in the last sentence of the paragraph is, therefore, incorrect, I believe.

We agreed with this point and revised the sentence accordingly. The text now states that no obvious cleavage was observed with the PE2/pegRNA condition, suggesting fewer editing events compared with PEn, rather than implying greater precision from the T7E1 result alone.

(4) Use of terminology. "Germline transmission" is typically used to refer to the fraction of F0s transmitting desired changes (or transgenes) to their progeny, while "germline mosaicism" refers to the fraction of F1s with the desired change in the progeny of a given F0. "Germline transmission" in line 217 should be replaced with "germline mosaicism".

We have replaced the terminology accordingly in the revised manuscript.

(5) Lines 253-255: The fraction of injected embryos that had mosaic nuclear expression of GFP, indicative of NLS insertion, should be clarified. It should also be clarified whether embryos positive for nuclear GFP were preselected for amplicon sequencing and germline transmission analyses. This is extremely important for extrapolation to scenarios like epitope tagging, where preselection is not possible.

We agree and have clarified this in the revised manuscript. We now state the fraction of injected embryos showing mosaic nuclear GFP expression, and we explicitly note that embryos were not preselected prior to sequencing or founder analysis. We further explain that preselection was not practical because the transgene is multicopy and individual fibres showed variable ratios of nuclear to cytoplasmic GFP, which made reliable scoring difficult.

(6) Statistical analyses. It would be helpful to clarify why different statistical tests are sometimes used to assess seemingly very similar datasets (Figures 1c, 1d, 2b, 2c, 2f).

We have clarified this in the Materials and Methods section and now state that the choice of statistical test depended on the normality and variance structure of the experimental data.

(7) Discussion. Since authors suggest that PEn might be especially beneficial for insertion of additional sequences, it is important to stress locus-to-locus variability of success. While the precise +3 insertion was indeed tremendously efficient at both tested loci (ror2 and adgrf3b), +12 addition into adgrf3b was over 10 times less efficient. In contrast, +30 into smyhc:GFP using the shorter pegRNA was highly efficient again. Longer pegRNA did not work nearly as well. As dangerous as it is to extrapolate from small datasets, perhaps these observations indicate that optimization of RT template and PBS may be needed for each new locus in order to significantly outperform oligonucleotide-mediated HDR? If so, would the cost of ordering several pegRNAs and the effort needed to compare them factor in when deciding which method to use?

We fully agree and have substantially revised the discussion to reflect this point. We now emphasise more clearly that editing efficiency is locus- and edit-dependent and likely influenced not only by insertion length but also by spacer sequence and pegRNA complexity. We cite the relevant literature on prime editing determinants and discuss that locus-specific optimisation may be required. We also softened our concluding claims so that the manuscript presents PEn as a practical donor DNA-free approach rather than as a universally high-efficiency solution.

**Recommendations for the authors:**

**Reviewer #1 (Recommendations for the authors):**

(1) Because this is a genome editing methods paper, including frequency or percentages of somatic and germline editing in the abstract, in comparison to previously published studies, it would be useful information for the intended audience

We agree and revised the abstract to include concrete editing frequencies. We now indicate the strongest insertion efficiencies observed. We also retained the statement that edited alleles were transmitted to the next generation.

**Reviewer #2 (Recommendations for the authors):**

(2) Please include additional loci for substitutions and insertions to strengthen conclusions about PE2/PEn efficiencies.

In response, we added further substitution data at the *ror2* (Figure 3—figure supplement 1) and insertion at the *wls* locus (Figure 5—figure supplement 2) and strengthened the presentation of the *adgrf3b* insertion data: first, by adding new locus data where feasible; and second, by narrowing the wording of our conclusions so that they are explicitly limited to the loci tested here.

(3) Please provide direct comparisons between zebrafish ror2 W722X phenotypes and human Robinow syndrome symptoms to support disease modeling claims.

We addressed this by adding analysis of the late *ror2* phenotype. In the revised manuscript, zygotic and maternal-zygotic mutants are reported to lack nasal and maxillary barbels, and one-year-old mutants show altered jaw morphology with a less protrusive lower jaw (Figure 4).

(4) The substitution of two nucleotides (+3 G→C and +10 A→G) to target residue I378 of crbn is not justified. It is unclear why two substitutions were required to model thalidomide sensitivity or validate editing efficiency. Please explain why dual nucleotide substitutions were necessary in the crbn experiments and whether single substitutions would suffice.

We now explain in the main text that the second substitution was introduced to reduce potential inhibitory intramolecular interactions within the pegRNA, while the primary substitution generated the intended amino-acid change. This clarification is now stated explicitly in the Results.

(5) The reported 10.3% precise editing efficiency for PEn/pegRNA at ror2 conflicts with Supplementary Figure 2, where none of the 20 clones from PEn/pegRNA showed precise edits, while one clone from PEn/springRNA did. Please address the inconsistency between NGS and cloning results at ror2, possibly by increasing sample size or reanalyzing sequencing data.

We addressed this directly by repeating and expanding the clone analysis. The revised Figure 2—figure supplement 1 now includes the updated clone dataset, and the result is in much better agreement with the NGS-based frequency estimates.

(6) Figure 3d highlights edits from PEn/springRNA but omits PEn/pegRNA results, despite the latter being described as superior. This creates ambiguity about the relative performance of pegRNA vs. springRNA. Please include PEn/pegRNA results in Figure 3d to fairly represent pegRNA performance.

We agree. We therefore revised Figure 3E so that it now includes alignment data for PE2/pegRNA, PEn/pegRNA and PEn/springRNA, allowing more direct visual comparison of the editing outcomes.

(7) The study does not specify the version of PEn used, or introduce some background of PE2 and springRNA. Comparisons to prior PE work in zebrafish, base editing, or HDR efficiencies are absent, obscuring the novelty of this approach. Please specify the PEn variant used, describe springRNA/PE2 structures, and compare results to prior zebrafish PE studies, BE, and HDR efficiencies for similar edits, contextualizing where PE2/PEn offers unique advantages.

We thank the editors for this helpful suggestion. We have clarified the PEn and PE2 systems in the manuscript, specified the nuclease-based PEn used, and improved the background text introducing these editing strategies. We added the data to directly compare prime editing and HDR in the *ror2* locus (Figure 3). We also expanded the Discussion to place the current findings in the context of prior zebrafish prime editing, HDR-based knock-in and base-editing work. We did not test all alternative systems experimentally in the current study, but we now discuss their relevance and clearly define the specific contribution of the present work.

(8) The manuscript does not explore advanced PE variants (e.g., PE3, PEmax), codon optimization, or scaffold modifications to improve efficiency. Please discuss whether codon optimization, PE3/PEmax systems, or pegRNA modifications were tested or could improve outcomes.

We agree that this should be discussed and we added recent work on zebrafish prime editing optimisation, codon optimisation, pegRNA engineering and related advances to the discussion, and explain that these are promising avenues for improving efficiency in future studies.

(9) No data compares the off-target effects of PE2 and PEn, a critical consideration for evaluating specificity and safety. Please perform comparative off-target analyses for PE2 and PEn to assess specificity.

In response, we performed comparative off-target analysis for the *ror2* target and analysed three predicted off-target sites. These data are now included in Figure 3—figure supplement 1 and show no significant increase in non-specific editing for the prime editing conditions tested.
